# Aberrant lncRNA and mRNA expression in patients with anti-*N*-methyl-d-aspartate receptor encephalitis

**DOI:** 10.3389/fneur.2025.1605982

**Published:** 2025-08-28

**Authors:** Xiaoyu Ma, Wenbo Guo, Zixuan Teng, Qingqing Yin, Chunjuan Wang, Pin Wang, Shougang Guo

**Affiliations:** ^1^Department of Neurology, The Second Qilu Hospital of Shandong University, Jinan, China; ^2^Laizhou No.1 Middle School, Yantai, Shandong, China; ^3^Department of Neurology, Shandong Provincial Hospital Affiliated to Shandong First Medical University, Jinan, Shandong, China; ^4^Department of Neurology, Shandong Provincial Hospital Affiliated to Shandong University, Jinan, Shandong, China

**Keywords:** anti-*N*-methyl-d-aspartate receptor encephalitis, long non-coding RNAs, messenger RNAs, RNA sequencing, neuroinflammation

## Abstract

**Background:**

Anti-*N*-methyl-d-aspartate receptor (anti-NMDAR) encephalitis is a nervous autoimmune disorder discovered in the recent more than 15 years. To understand potential involvement of epigenetic mechanisms in pathogenesis of anti-NMDAR encephalitis, we initiated a study to compare the expression profiles of long non-coding RNAs (lncRNAs) and messenger RNA (mRNAs) in patients of anti-NMDAR encephalitis and healthy controls.

**Methods:**

Eleven patients who were diagnosed with anti-NMDAR encephalitis were enrolled in our observational studies. Total RNA was extracted from patients’ plasma and the expression levels of lncRNAs and mRNAs were determined. Differential expression analysis via RNA sequencing, Gene Ontology (GO) and Kyoto Encyclopedia of Genes and Genomes (KEGG) pathway analysis, as well as a co-expression network of lncRNA-mRNA were performed in 5 patients and 5 healthy controls to evaluate potential the changes in expression patterns. The expression levels of certain lncRNAs and mRNAs were further validated in 11patients and 11 healthy controls using quantitative real-time polymerase chain reaction (qRT-PCR).

**Results:**

It was found that a total of 83 lncRNAs and 2,345 mRNAs were differentially expressed in five patients with anti-NMDAR encephalitis compared with five healthy controls. Of those lncRNAs, 63 were upregulated and 20 downregulated, while 1,509 mRNAs were upregulated and 836 downregulated. GO and KEGG pathway analyses showed that a wide range of biological functions were perturbed during acute anti-NMDAR encephalitis. Differentially expressed genes were found to be involved in autoimmune, B cell signaling, neuroinflammation, or synaptic plasticity. qRT-PCR was conducted in 11 patients and 11healthy controls to further confirm the levels of six lncRNAs and four mRNAs, and the results were found to be consistent with those by RNA sequencing. The co-expression networks of lncRNA-mRNA were performed in some meaningful KEGG analyses, including chemokine signaling pathway, Long-term potentiation, B cell receptor signaling pathway and MAPK signaling pathway.

**Conclusion:**

Taken together, these findings suggest the involvement of a number of molecular pathways in anti-NMDAR encephalitis, some of which could serve as potential biomarkers to assist in diagnosis, treatment and prognosis.

## 1 Background

The past decade has observed much better understanding and improved care of anti-N-methyl-D-aspartate receptor (anti-NMDAR) encephalitis. The disease is now known to present with the subacute development of memory or behavioral deficits, alteration in consciousness, psychiatric symptoms, speech dysfunction, autonomic dysfunction and often seizures (1). There is also a female sex predominance with the disease, along with accompany of ovarian teratoma (2). Currently, the autoantibody testings are not readily available at disease onset and response to immunotherapy might have late effect and patients with other disorders can also respond to therapy, which can lead to misjudge its severity and even misdiagnosis. Auxiliary examinations such as brain MRI, EEG and lumbar puncture are helpful but only as non-specific references, in contract to more desirable objective and specific diagnosis. Moreover, pathogenic mechanisms remain to be clarified and subject to further research efforts (3).

The majority of studies in the field have so far focused on protein interactions and expression (such as antibodies and cytokines, etc.) (4, 5), and only limited attention has set on non-coding RNAs (ncRNAs), such as long non-coding RNAs (lncRNAs), micro RNAs (miRNAs), and circular RNAs (circRNAs). Recently, the differential miRNA expression profiles of anti-NMDAR encephalitis were established by a microarray approach, and 66 differentially expressed miRNAs were identified. Furthermore, after comparison with some other central nerve system diseases, it was suggested that let-7b may be a potential diagnostic indicator and a treatment biomarker (6). lncRNAs, as non-protein coding RNAs of longer than 200 nucleotides in length, have increasingly attracted researchers’ attention and are now implicated in pathological processes and biological functions of diverse immune disorders, reportedly by increasing or preventing transcription and by modifying chromatin structure (7). There exists the prospect that lncRNAs are utilized as potential biomarkers to assist in diagnosis, treatment and prognosis. For anti-NMDAR encephalitis, the involvement of lncRNAs remains to be ascertained and a reference database is lacking.

We therefore initiated this study to investigate the differential expression of lncRNAs that potentially associates with anti-NMDAR encephalitis by RNA sequencing (RNA-seq). Quantitative real-time polymerase chain reaction (qRT-PCR) was used to confirm the differentially expressed patterns of selected lncRNAs and mRNAs. Additionally, we performed gene ontology (GO) and Kyoto Encyclopedia of Genes and Genomes (KEGG) pathway analyses and established a lncRNA-mRNA co-expression network. To our knowledge, this is the first comprehensive analysis on lncRNAs in anti-NMDAR encephalitis and hopefully helps understand this disorder at the RNA level.

## 2 Methods

### 2.1 Patients and sample collection

This study enrolled 11 patients, who had been diagnosed with definite anti-NMDAR encephalitis and admitted to Shandong Provincial Hospital affiliated to Shandong University and The Second Hospital of Shandong University. The diagnosis was based on criteria proposed by Graus et al. (1). NMDAR antibodies were detected in both cerebrospinal fluid and blood using transfected human embryonic kidney cells (HEK293) expressing NR1 subunits of the NMDA receptor by third-party medical testing agencies. The blood samples from these patients were collected in their acute period prior to administration with glucocorticoid or intravenous immune globulin. Another 11 healthy people with matching age and gender were selected as the contrast group. Their demographic features are summarized in Table 1. Fresh fasting peripheral blood samples were acquired in tubes with ethylene diamine tetra acetic acid (EDTA) early in the morning, followed by centrifugation to obtain fresh plasma at 1,600 × g for 10 min at 4 °C. Subsequently, the plasma was stored at −80 °C.

**Table 1:** Demographic features of patients with anti-NMDAR encephalitis and controls.

Characteristics	Patients (*N* = 11)	CTLs (*N* = 11)	*P*-value
Age, years (median, range)	23 (14–53)	23 (14–53)	1
Female (*n*, %)	7 (63.6)	7 (63.6)	1
mRS score			
At admission (median, range)	5 (3–5)	–	–
At 3 months after discharge (median, range)	1 (0–3)	–	–
Clinical symptoms (*n*, %)		–	–
Prodromal symptoms (fever, headache)	3 (27.3)	–	–
Cognitive disorder	5 (45.5)	–	–
Psychiatric and behavioral abnormalities	7 (63.6)	–	–
Speech disorder	4 (36.4)	–	–
Seizures	6 (54.5)	–	–
Disturbance of consciousness	5 (45.5)	–	–
Central hypoventilation	2 (18.2)	–	–
Involuntary movement	3 (27.3)	–	–
Tumor presence (*n*, %)	3 (27.3)	–	–
CSF antibody positivity (*n*, %)	11 (100)	–	–
Serum antibody positivity (*n*, %)	8 (72.7)	–	–

Values are presented as numbers (%), or medians (range). CTLs, controls; mRS, Modified Rankin Scale; CSF, cerebrospinal fluid.

### 2.2 RNA extraction

Total RNA was extracted from stored plasma using RNeasy mini kit (Qiagen, Germany). RNA purification was done using a DNase method following the manufacturer’s instructions. Sample quality and quantification were further confirmed by an Agilent Bioanalyzer 2,100 (Agilent technologies, United States).

### 2.3 Library construction and sequencing

The samples were then submitted to Shanghai Biotechnology Corporation for further library construction and sequencing. Briefly, the strand-specific libraries construction was performed using rRNA-depleted RNAs with TruSeq^®^ Stranded Total RNA Sample Preparation kit (Illumina, USA) according to the manufacturer’s instructions. Using Ribo-Zero rRNA removal beads, ribosomal RNA was removed from total RNA. Following purification, the mRNA was then broken into small fragments by incubating with divalent cations for 8 min at 94°C. Then, the cleaved RNA pieces are copied into first strand cDNA through reverse transcriptase and random primers. Second strand cDNA was synthesized by using DNA Polymerase I and RNase H. Afterwards, the single ‘A’ bases was added to these cDNA fragments for end repair, which was followed by ligation of the adapters. The cDNA library was eventually created after the products were purified and enriched, and then quantified by Qubit^®^ 2.0 Fluorometer (Life Technologies, United States) and validated by Agilent 2,100 bioanalyzer (Agilent Technologies, United States). Cluster was generated by cBot with the library diluted to 10 pM and then were sequenced on the HiSeq^®^ 2,500 System (Illumina, United States).

### 2.4 Data analysis for gene expression profiling (RNA-seq)

Whole transcriptome sequencing data were filtered by removing sequencing adapters, rRNA reads, short-fragment reads and other low-quality reads. The trimmed data were then mapped to the human hg38 reference genome with two mismatches using Tophat v2.0.9 (8). After genome mapping, we used Cufflinks v2.1.1 (9) with a reference annotation to generate fragments per kilobase of exon model per million mapped reads (FPKM) values for known gene models and then evaluate the differentially expressed lncRNAs and mRNAs level. According to the FPKM, the fold change was determined. False discovery rate (FDR) was applied based on the *p*-value significance threshold. The defining criteria of differentially expressed lncRNAs and mRNAs were two folds: FDR less than 0.05 and fold change greater than 2.0.

### 2.5 lncRNAs identification and expression analysis

We acquire novel transcripts which were first assembled from reads using Cufflinks and then compared with the human known protein coding transcripts using Cuffcompare (9). Novel transcripts with length ≥200 bp, ORF ≤300 bp, no or weak protein coding ability, number of exons ≥ 2, were defined as putative lncRNAs. Subsequently, we used Cuffcompare to integrate the RNA-seq derived lincRNAs with the known lncRNAs previously annotated by NONCODE v4 to extract different lncRNAs in anti-NMDAR encephalitis. Target prediction which made according to differentially expressed lncRNAs can be composed of two categories: *cis*-acting target genes and trans-acting target genes. The former were genes transcribed within a 10 kbp window upstream or downstream of lncRNAs while the latter were predicted using RNAplex software (10).

### 2.6 GO and KEGG analysis

Functional classification of differentially expressed mRNAs was performed by GO analysis, which can characterize genes according to three aspects: cellular components, molecular functions, and biological processes. The enriched biological KEGG analysis was identified to discover the main pathways in the differentially expressed mRNAs and predict the underlying functions.

### 2.7 Co-expression analysis

The lncRNA-mRNA co-expression analysis was based on calculating statistically significant associations through Pearson’s correlations between lncRNAs and mRNAs whose expression levels shared a meaningful correlation.

### 2.8 qRT-PCR validation

Six differentially expressed lncRNAs (5 upregulated lncRNAs including ENST00000569449, ENST00000400593, ENST00000525556, ENST00000430776, NONHSAT171861.1, 1downregulated lncRNA NONHSAT212407.1) and four differentially upregulated mRNAs (GUCY1A3, IDS, PARVB, TROVE2) were selected to confirm the sequenced expression data by the qRT-PCR assay. Total RNA was isolated using RNeasy mini kit (Qiagen, Germany) according to following the manufacturer’s protocol. Purified total RNA was subsequently reverse transcribed using the ReverTra Ace qPCR RT Kit (TOYOBO, China) following the manufacturer’s instructions. Real-time qPCR was performed in a Cycler (Bio-Rad, United States) using UltraSYBR Mixture (CWBIO, China). The expression levels of the genes of interest were normalized by using Glyceraldehyde phosphate dehydrogenase (GAPDH) as internal standard with an average of three pores and the PCR results were quantified by the 2-∆∆ct method. The primers are as follows: ENST00000569449 forward primer, TTTTACTTGGGAAACTGCTAC, reverse primer, TATGGTACTTCACTAGGGTGG, ENST00000400593 forward primer, CGAGTATTTGTAGAAGGGTATGGTT, reverse primer, ACGGGCGACTGTAGTGTTGTT, ENST00000525556 forward primer, CTTCACCTGGGCTGCATAGCT, reverse primer, TCTTTCCCTCTTTCTTTCTTCTTC, ENST00000430776 forward primer, TCAGTTTCTGTGGGAGGGTAG, reverse primer, GCAGGTTCTTCAGGAGGTATT, NONHSAT171861.1 forward primer, GTTTGAGCGATCCCTCTATCC, reverse primer, TCCACATATCAGCCACTTCCA, NONHSAT212407.1 forward primer, GGTGCAACTCCTCTTACCTAG, reverse primer, GTCGCATCTTTATTCCAATCT, GUCY1A3 forward primer, TGAAATGCTGCCAAATCCATC, reverse primer, TGCTGCTCTTGTTCCAGGTGT, IDS forward primer, CGCTTATCCATTCATCCACCA, reverse primer, GTTACCTCCCAGCACAAACCC, PARVB forward primer, TCAAGAAACCCAAGGCTCGTC, reverse primer, ACACTCCTGCCACCATCCACA, TROVE2 forward primer, GTTTGTCGCATTCCTACCCAT, reverse primer, TCTTTGTGAGACCAGCCATTT.

### 2.9 Statistical analysis

Statistical analyses were performed using SPSS version 24.0 (SPSS Inc., United States). The *χ*^2^ test was used for categorical variables. The student’s *t*-test was analyzed for continuous variables such as validation of differentially expressed representative lncRNAs and mRNAs in anti-NMDAR encephalitis compared to healthy controls. Pearson’s correlations were performed to analyze the lncRNA-mRNA co-expression associations. *p*-values less than 0.05 were considered significant.

## 3 Results

### 3.1 Differential expression of lncRNA and mRNA profiles in patients with anti-NMDAR encephalitis and healthy controls

**Figure 1:**
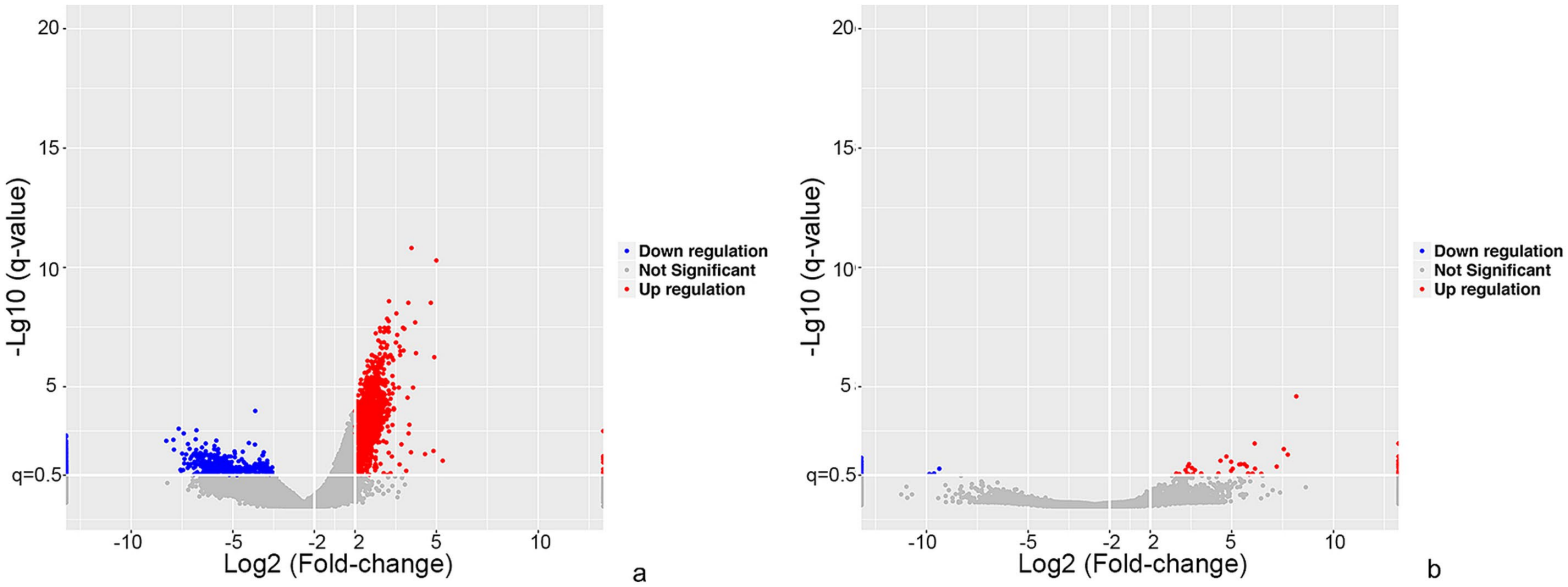
Overview of differentially expressed mRNA and lncRNA profiles between patients with anti-NMDAR encephalitis and controls. The visualization of volcano plots showed mRNAs **(a)** and lncRNAs **(b)**. The vertical lines represent two-fold up and down and horizontal lines correspond to a *p*-value of 0.05. Red points: upregulated mRNAs or lncRNAs, blue points: downregulated mRNAs or lncRNAs.

**Figure 2:**
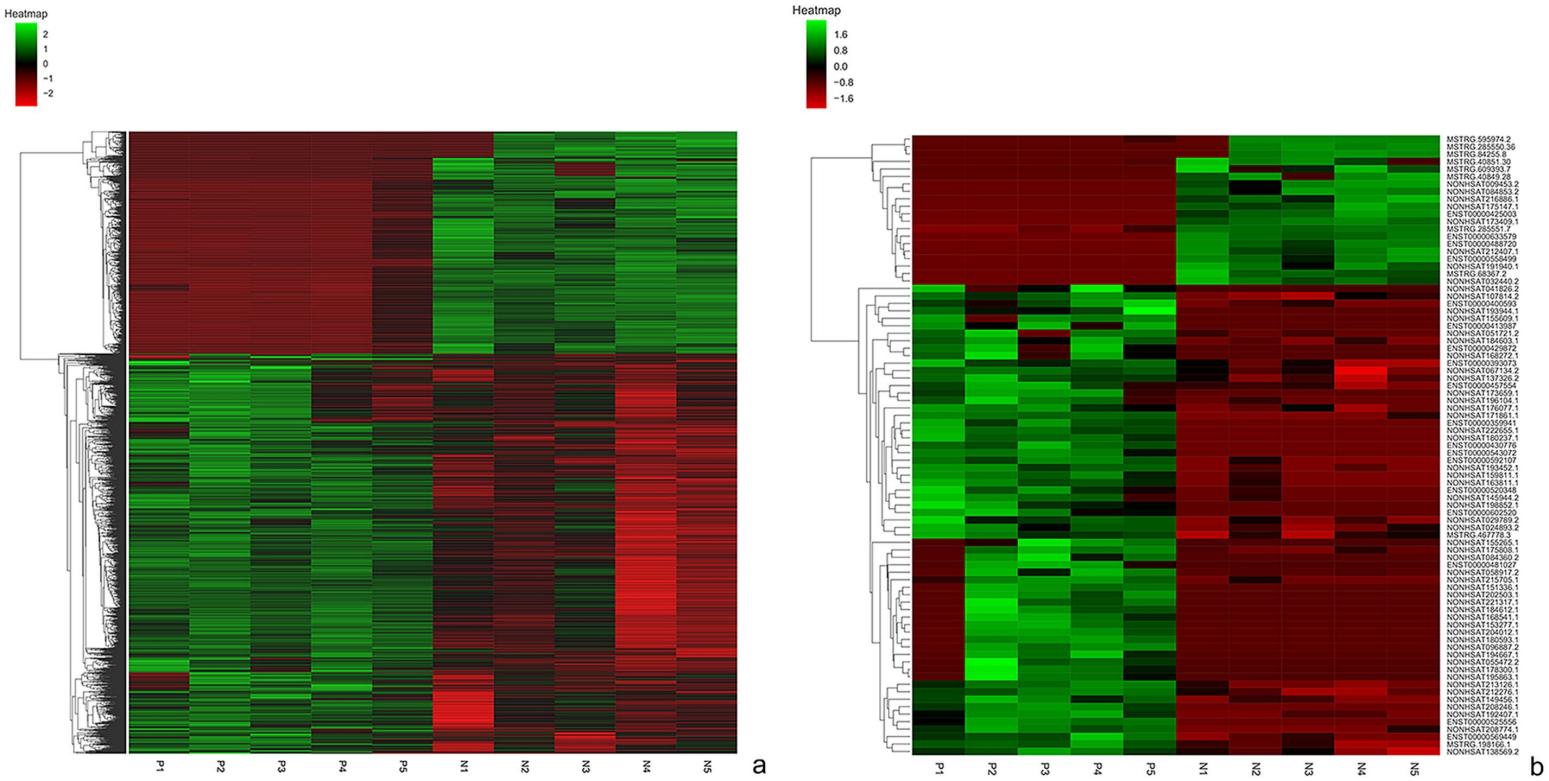
Hierarchical clustering of mRNAs **(a)** and lnRNAs **(b)** in patients with anti-NMDAR encephalitis and controls. P represents patients with anti-NMDAR encephalitis, C represents controls.

**Figure 3:**
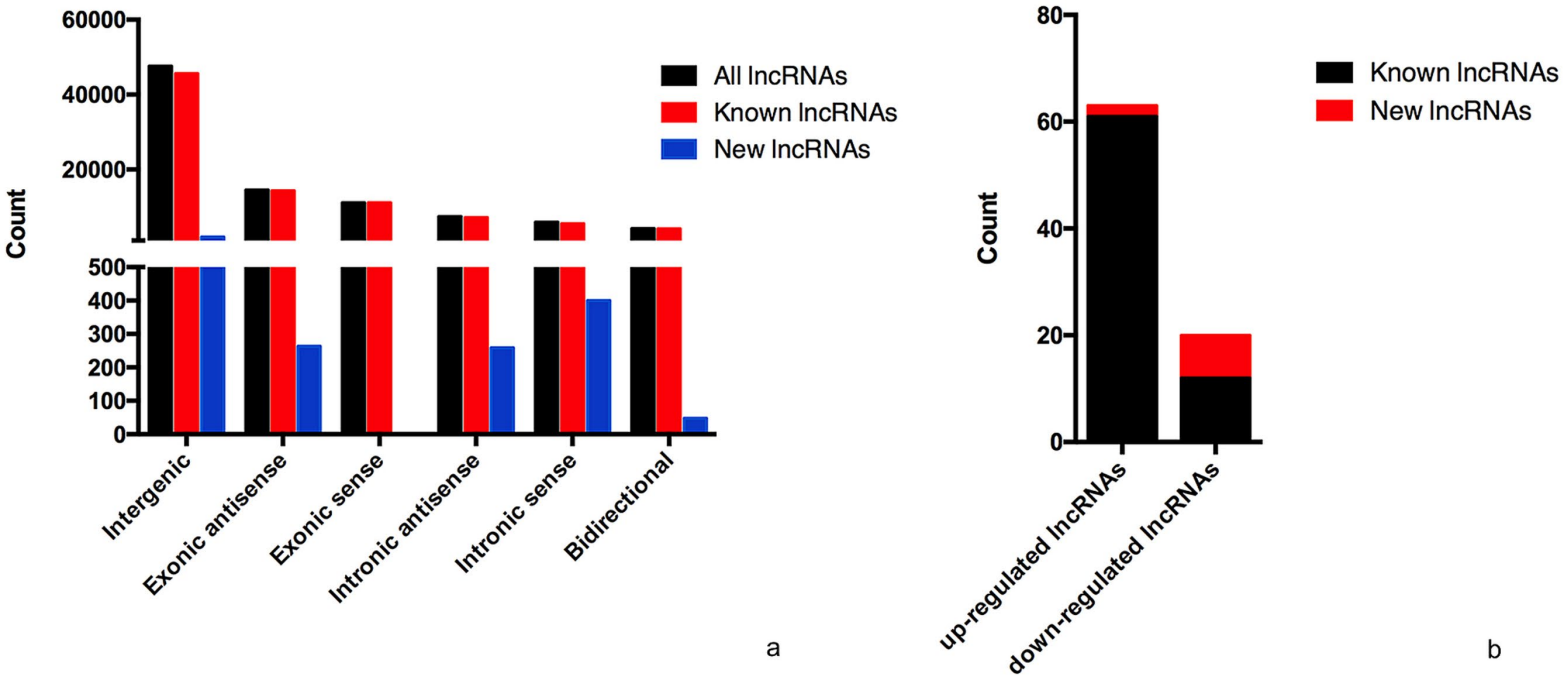
General characteristics of lncRNAs in patients with NMDAR encephalitis compared to controls. **(a)** The categories of all lncRNAs. **(b)** Classification of differentially expressed lncRNAs.

The lncRNA and mRNA profiles were determined from five patients with anti-NMDAR encephalitis and five healthy controls. A total of 90,967 lncRNAs were evaluated, including 88,034 lncRNAs known in NONCODE database^1^ and the Ensembl database,^2^ 2,933 lncRNAs that were first identified in this study. We then applied volcano plot analysis to identify the different expression of lncRNAs and mRNAs among samples (Figure 1). In addition, hierarchical clustering was used to distinguish patients with anti-NMDAR encephalitis from controls (Figure 2). Based on the gene expression profiling data, 83 lncRNAs were found to be specifically dysregulated with a 2-fold change as the cutoff, of which 63 lncRNAs were upregulated, and 20 lncRNAs downregulated, respectively (Figures 1b, 2b). Additionally, distinguishable mRNA expression patterns from these two populations were also showed by hierarchical clustering in Figure 2a, a total of 2,345 mRNAs were found to be differentially expressed, including 1,509 upregulated mRNAs and 836 downregulated mRNAs (Figures 1a, 2a). The general characteristics of lncRNAs in patients with NMDAR encephalitis compared to controls are shown in Figure 3.

### 3.2 GO and KEGG enrichment analysis

**Figure 4:**
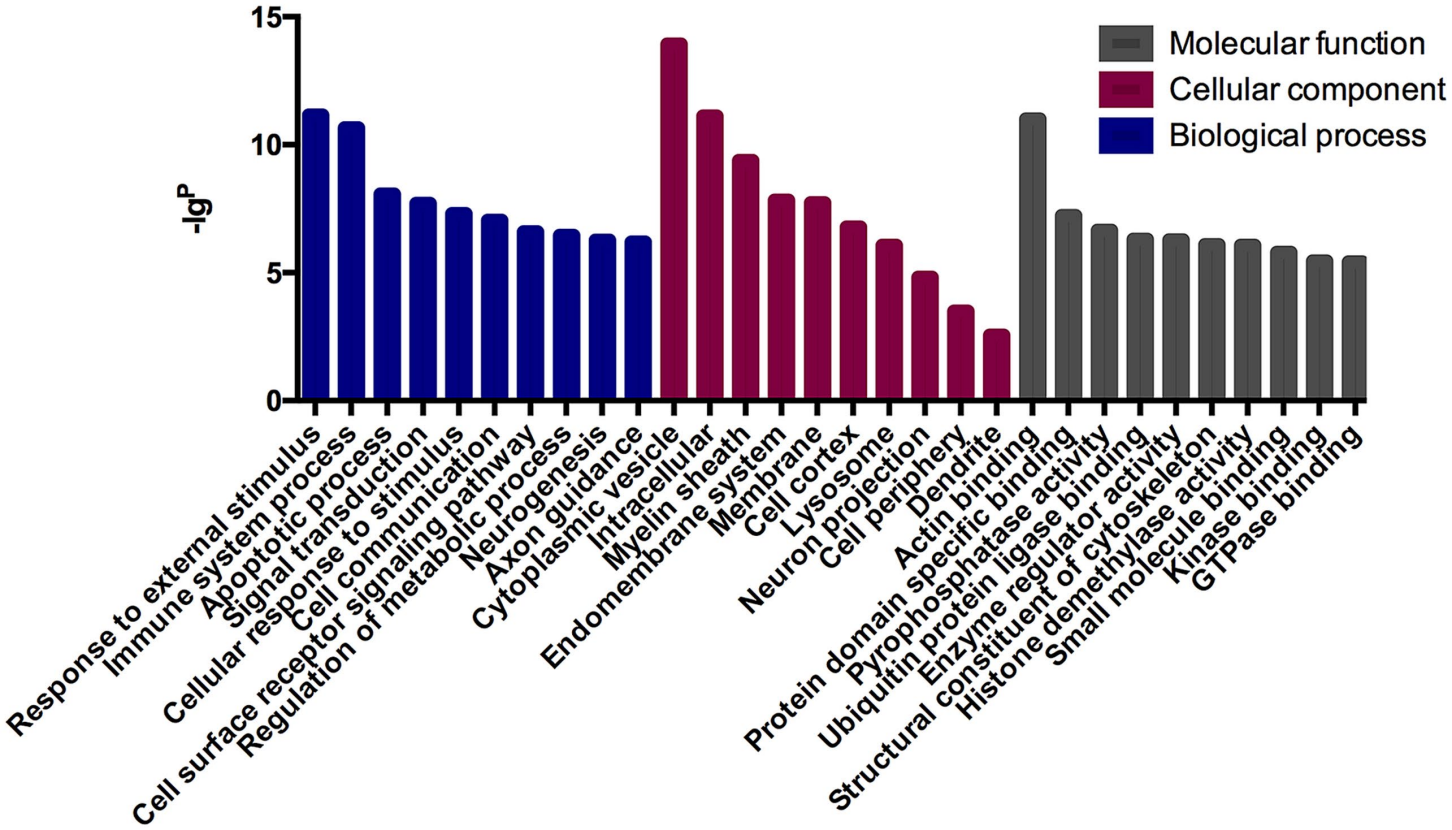
GO analysis in anti-NMDAR encephalitis compared with healthy controls. The x-axis represents annotation of the aberrant enriched mRNAs covering domains of biological processes, cellular components, and molecular functions. The *y*-axis represents enrichment score which showed as the –lg*p* value.

**Figure 5:**
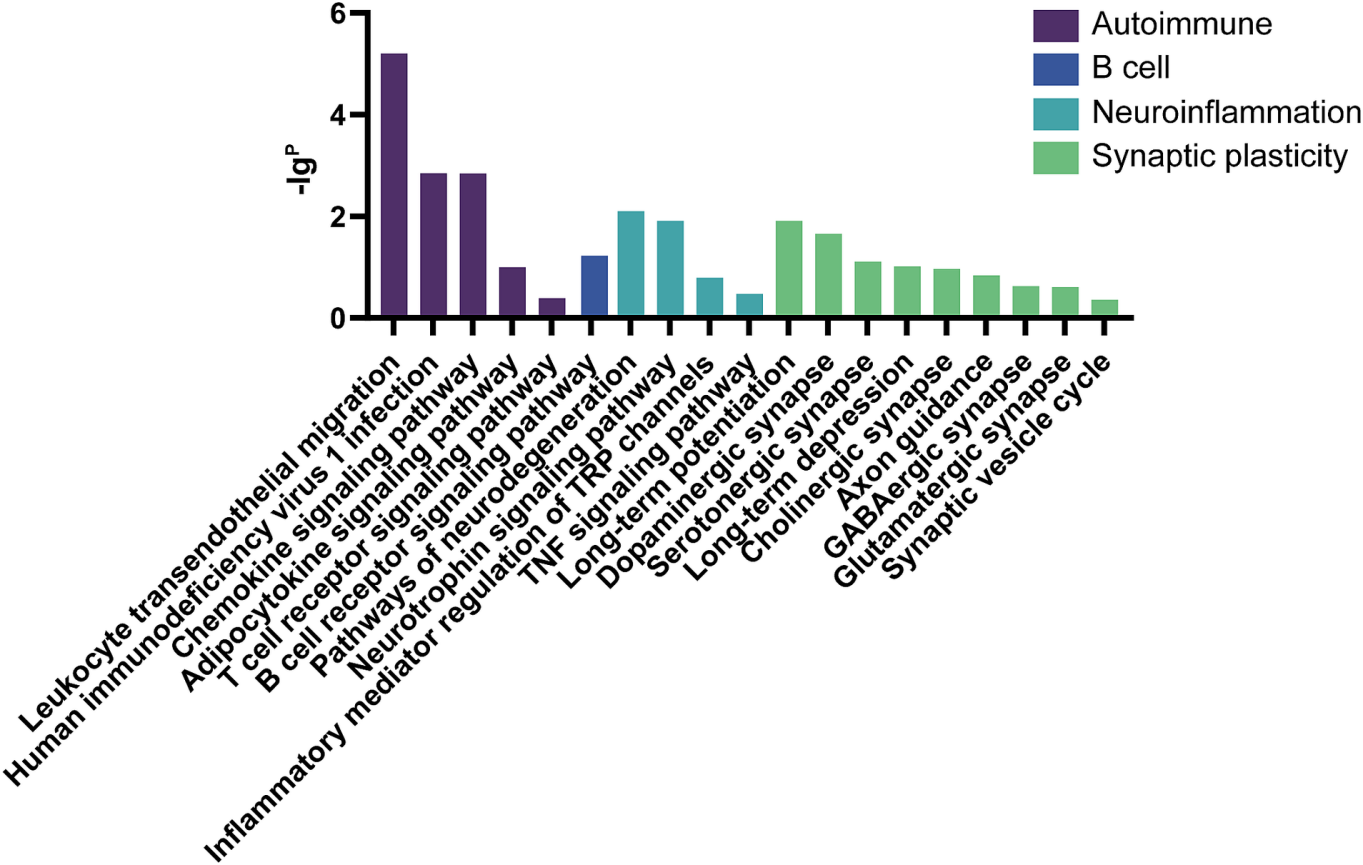
KEGG analysis in anti-NMDAR encephalitis compared with healthy controls. The *x*-axis represents annotation of the aberrant enriched mRNAs. The *y*-axis represents enrichment score which showed as the –lgP value.

The GO analysis was conducted to evaluate the enrichment of the differentially expressed mRNAs in cellular components, molecular functions, and biological processes. The GO analysis results showed that the enriched GO terms of the dysregulated aberrantly mRNAs include response to external stimulus, immune system process, apoptotic process, signal transduction, cellular response to stimulus in BP, cytoplasmic vesicle, intracellular, myelin sheath, endomembrane system in CC, actin binding, protein domain specific binding, pyrophosphatase activity, ubiquitin protein ligase binding, enzyme regulator activity in MF, respectively (Figure 4). KEGG enrichment analysis selected that the pathways of dysregulated mRNAs were associated with autoimmune, B cell signaling, neuroinflammation, or synaptic plasticity (Figure 5). Significantly altered genes based on KEGG pathway enrichment are shown in the Table 2.

**Table 2:** Significantly altered genes based on KEGG pathway enrichment.

Theme	Pathway name	Genes
Autoimmune	Leukocyte transendothelial migration	ITGB1, ROCK1, NCF2, ROCK2, NCF4, PIK3CB, CD99, CD146
	Human immunodeficiency virus 1 infection	ITPR2, PIK3CB, GNAI2, GNG10, PPP3R1, GNG3, AP1S2
	Chemokine signaling pathway	ROCK1, CXCL13, ADCY2, ARRB2, PIK3CB, CXCL5, GNAI2
	Adipocytokine signaling pathway	CPT1A, ACSL1, STAT3, IRS4, ACSL4, PTPN11, ADIPOR1
	T cell receptor signaling pathway	NFATC3, PIK3CB, MAPK14, RHOA, NFKBIA, CDC42, PPP3R1
B cell	B cell receptor signaling pathway	LYN, IFITM1, PRKCB, NFATC3, DAPP1, PIK3CB, NFKBIA
Neuro-inflammation	Pathways of neurodegeneration	CHRM3, APP, ATP2A3, PARK7, COX7C, GRM1, UBE2L3
	Neurotrophin signaling pathway	YWHAE, IRAK3, PTPN11, PIK3CB, MAPK14, RHOA, RAP1B
	Inflammatory mediator regulation of TRP channels	MAP2K3, PRKCB, ITPR2, TRPV3, ALOX12, ADCY2, PIK3CB
	TNF signaling pathway	MAP2K3, ATF2, VEGFC, PIK3CB, MAPK14, CXCL5, NFKBIA
Synaptic plasticity	Long-term potentiation	PRKCB, ITPR2, GRM1, PPP1CA, PPP1CB, RAP1B, PPP3R1
	Dopaminergic synapse	ATF2, MAOB, ITPR2, ARRB2, GNAI2, PPP1CB, GNG10
	Serotonergic synapse	APP, MAOB, PRKCB, DUSP1, ITPR2, ALOX12, GNG11
	Long-term depression	GNA13, LYN, GNAZ, PRKCB, GNAQ, GNAS, ITPR2
	Cholinergic synapse	CHRM3, PRKCB, ITPR2, ADCY2, PIK3CB, GNG11, GNAI2
	Axon guidance	ROCK1, ROCK2, ILK, PIK3CB, MYL12A, MYL12B
	GABAergic synapse	GABARAPL2, PRKCB, ADCY2, GNG11, GABARAP, GNAI2, TRAK2
	Glutamatergic synapse	PRKCB, ITPR2, ADCY2, GNG11, GRM1, GNAI2, GNG10
	Synaptic vesicle cycle	DNM3, ATP6V1A, NAPA, ATP6V1G1, ATP6V0E1, AP2B1, ATP6V1E1

### 3.3 qRT-PCR validation

**Figure 6:**
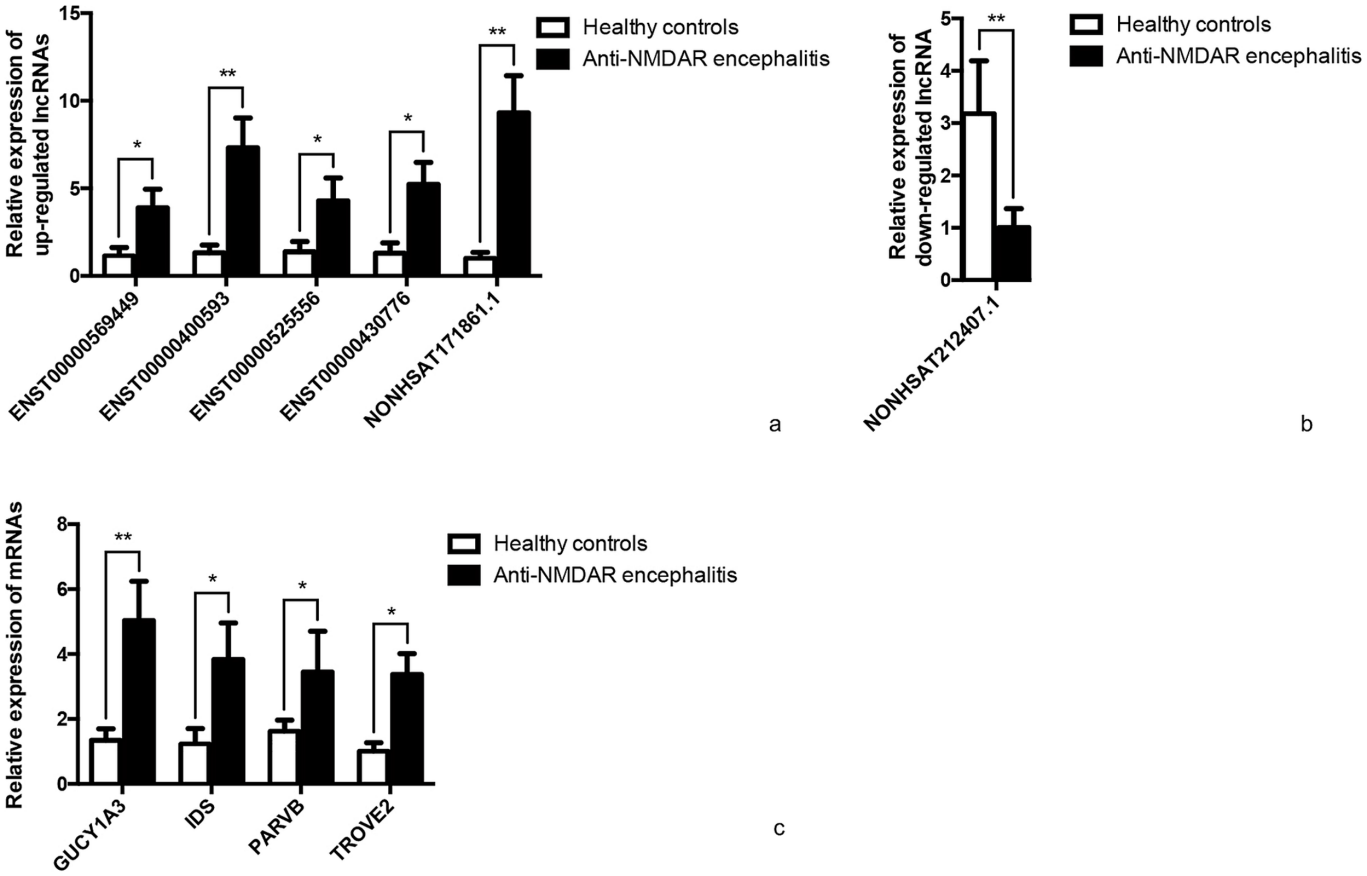
qRT-PCR validation. Differentially expressed representative lncRNAs and mRNAs were validated in 11 anti-NMDAR encephalitis and 11 healthy controls normalized to GAPDH. **(a)** Relative expression of up-regulated lncRNAs. **(b)** Relative expression of down-regulated lncRNA. **(c)** Relative expression of mRNAs. *p < 0.05, **p < 0.01.

Five candidate lncRNAs from 63 upregulated lncRNAs, one from 20 downregulated lncRNAs, and four mRNAs were selected to further confirm the sequencing data in extension 11 plasma samples of from patients with anti-NMDAR encephalitis and controls by qRT-PCR, and the results were consistent with the sequencing data (Figure 6).

### 3.4 Construction of the lncRNA-mRNA co-expression network

**Figure 7:**
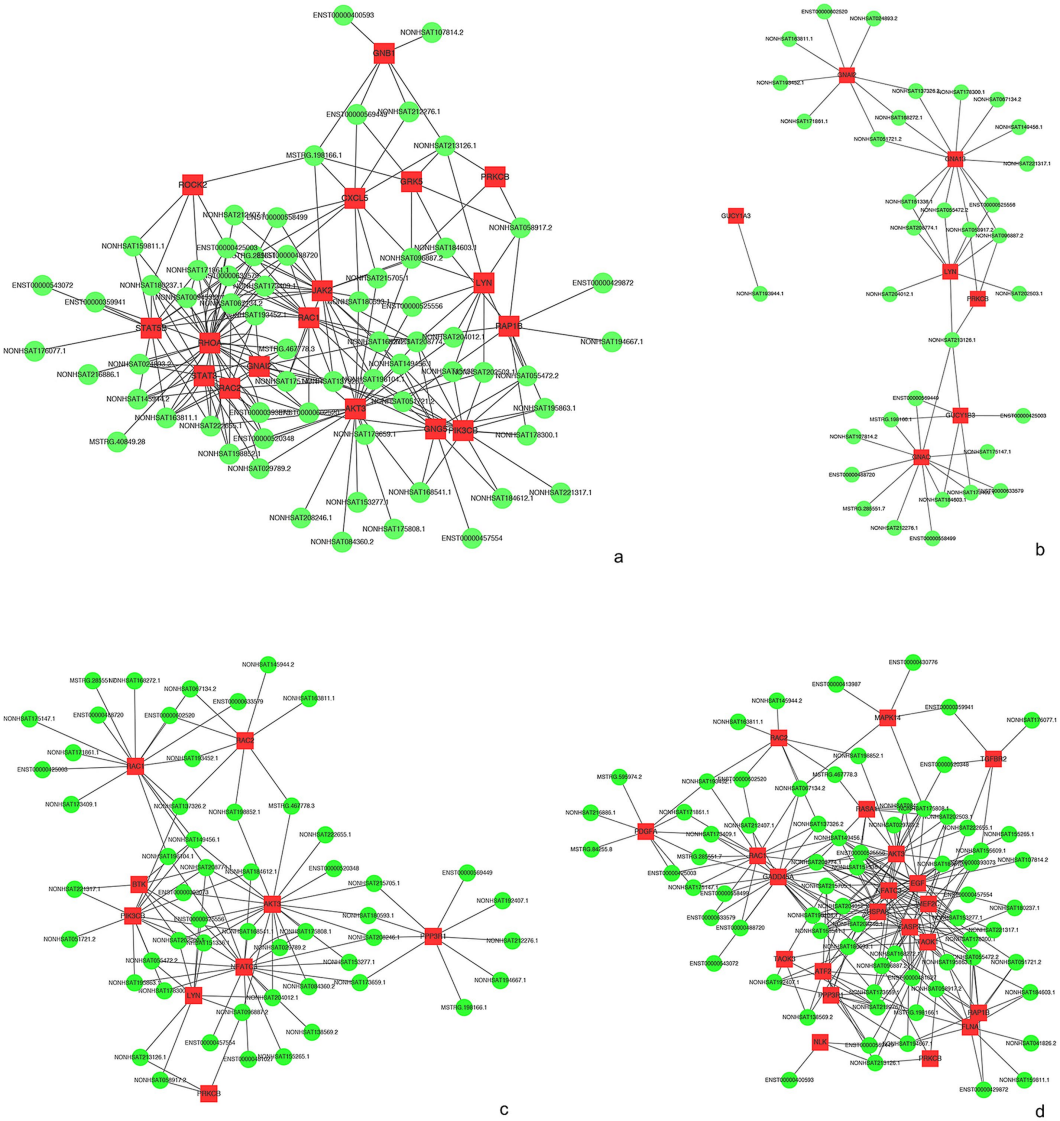
IncRNA-mRNA network analysis. **(a)** KEGG of “chemokine signaling pathway.” **(b)** KEGG of “Long-term potentiation.” **(c)** KEGG of “B cell receptor signaling pathway’. **(d)** KEGG of “MAPK signaling pathway.” Red and green colors represent mRNAs and lncRNAs, respectively.

The complex functions of the vast lncRNAs are still a matter of further understanding. Nonetheless, through the annotations with the co-expressed mRNA function, we are able to forecast the function of dysregulated lncRNAs to some extent. Our data showed that the co-expression network was composed of 83 differentially expressed lncRNAs together with 1,683 aberrantly expressed mRNAs. We constructed the lncRNA-mRNA co-expression network in some meaningful KEGG analyses. As we can see in Figure 6, 64 lncRNAs interacted with 17 mRNAs in the chemokine signaling pathway (Figure 7a), 34 lncRNAs interacted with 7 mRNAs in long-term potentiation (Figure 7b), 53 lncRNAs interacted with 9 mRNAs in the GO of B cell receptor signaling pathway (Figure 7c), and 71 lncRNAs interacted with 21 mRNAs in MAPK signaling pathway (Figure 7d). Taken together, the lncRNA-mRNA co-expression network indicates that these lncRNAs play important roles during the process of anti-NMDAR encephalitis.

## 4 Discussion

Anti-NMDAR encephalitis, an autoimmune and inflammatory disorder first recognized by Josep Dalmau in 2007, usually presents with a rapid progression of neuropsychiatric manifestations that may include memory declination, seizures, aggressive behaviors, speech disorder, emotion changes, autonomic dysfunction, and even decreased level of consciousness. It is associated with IgG antibodies against the NMDAR receptor subunit GluN1 (11). NMDA receptors are subtypes of glutamate receptors and are closely related to synaptic plasticity, learning and memory. Some patients could be misdiagnosed and admitted to psychiatric centers due to predominant psychiatric symptoms.

Various inflammatory cytokines/chemokines, such as IL-6, IL-17A, CXCL13 in cerebrospinal fluid, and IL-2 in plasma, and miRNAs (like e.g., Let-7b) in plasma, have been shown to play important roles in the process of anti-NMDAR encephalitis (5, 6, 12). Ideally, biomarkers used to diagnosis should be easily accessible, identifiable and quantifiable. Furthermore, researchers have concluded that regulation at both transcriptional and post-transcriptional levels can influence the behaviors of immune cells (B cells, T cells, macrophages, and NK cells) and the expression of inflammatory cytokines (6, 13, 14). Therefore, it is of interest to explore if such differential expression patterns of lncRNAs play a role in anti-NMDAR encephalitis. However, to the best of our knowledge, the involvement of lncRNAs in anti-NMDAR encephalitis has not been delineated. Here, we report the differential expression profiles of human lncRNAs and mRNAs in patients with anti-NMDAR encephalitis in comparison with healthy controls. In this study, we first compared 5 test samples and 5 control samples via RNA-seq and identified differentially expressed mRNAs (1,509 upregulated and 836 downregulated) and lncRNAs (63 upregulated and 20 downregulated). Subsequently, five upregulated lncRNAs (ENST00000569449, ENST00000400593, ENST00000525556, ENST00000430776, and NONHSAT171861.1), one downregulated lncRNA (NONHSAT212407.1) and four upregulated mRNAs (GUCY1A3, IDS, PARVB, TROVE2) were selected for additional assessment by qRT-PCR in 11 test samples and 11 control samples. The results were found to be consistent with the sequencing data. Consequently, we have come to the realization that certain lncRNAs and mRNAs in plasma may correlate with the development of anti-NMDAR encephalitis, and therefore could serve as a class of promising biomarkers for diagnosis and possibly new therapeutic targets to treat the disorder.

With the discovery of functions for several lncRNAs, such as H19, Xist, HOTAIR, it is now believed that these non-coding protein segments are genetic elements that participate in important biological functions through diverse mechanisms, rather than “transcriptional noise” (7). Most lncRNAs are transcribed by RNA polymerase II, structurally similar to mRNAs, and undergo 5′ capping and polyadenylation (15). Surprisingly, unlike protein-coding genes requiring transcription and translation to the final product protein to exert their biological function, lncRNAs can fold into high order structures and directly exert their functions without the process of translation (16). Recently, a series of reports have implicated the involvement of lncRNAs in autoimmune and inflammatory disorders, such as multiple sclerosis (MS), neuromyelitis optica (NMO), SLE, rheumatoid arthritis (RA), and autoimmune thyroid disease (14, 17–20). Similarly, lncRNAs were reported to participate in the development and progression of the neurodegenerative diseases, cancer, temporal lobe epilepsy, and stroke (21–24). Previous studies have reported CXCL13 and CD146 as potential biomarkers for anti-NMDAR encephalitis (25). In our analysis, while we also observed changes in these two genes, we additionally identified other members of the same families—CXCL5 and CD99—which may serve as novel biomarkers and provide additional reference value for diagnosis.

Our results from GO and KEGG analysis, as shown in figures above, revealed several interesting differential expression profiles. GO enrichment analysis suggested that dysregulated mRNAs mainly associate with cellular responses to external stimulus, immune responses, apoptotic processes, signal transduction, cytoplasmic vesicle, as well as processes involving myelin sheath, actin binding, protein domain specific binding, pyrophosphatase activity, ubiquitin protein ligase binding. Furthermore, the KEGG analysis implicated mRNAs whose aberration has been found to associate with viral carcinogenesis, focal adhesion, MAPK signaling pathway, pathways in cancer, apoptosis, as well as inflammation, immunity and degenerative nervous diseases. The current findings suggest possible scenarios where tumorigenic events (especially ovarian teratoma) or viral infections (mostly herpes simplex encephalitis) could contribute to the pathogenesis of anti-NMDAR encephalitis (26). There is a general consensus on the intertwining relation between immune response and tumorigenesis, and it is tempting to speculate that similar mechanisms could exist for anti-NMDAR encephalitis as well. Interestingly, the presence of nervous tissue was demonstrated in the patients’ tumor whether they are with or without anti-NMDAR encephalitis, whereas inflammatory infiltrates were found more extensive in the former (26). Previous studies also indicated a possible link between central nervous system (CNS) infections and anti-NMDAR encephalitis. Of note, this might be consistent with our KEGG analysis regarding viral carcinogenesis, pathways in cancer, and those related to inflammation and immunity.

Among them, the MAPK pathway including ERK2 is one of the most ubiquitous signal transduction cascade, as it occupies a pivotal role in a molecular signaling network in regulating cell growth, differentiation, proliferation, apoptosis and inflammation (27). Growing evidence shows the dysregulation of this pathway is common in various diseases, ranging from degenerative diseases (such as Alzheimer’s disease) to inflammatory diseases (such as MS), cancer, and autoimmune disorders (28–30). Through a cuprizone-induced demyelination model, Okazaki et al. observed that ablating the function of Erk2 in the CNS attenuated immune mediators and demyelination (29). Because inflammatory stimuli activates MAPK pathways to induce inflammatory responses, MAPK inhibitors emerge as promising candidates of effective anti-inflammatory drugs (31, 32). Interestingly, GNB4, a gene encoding a beta subunit in the heterotrimeric G proteins that, plays a crucial role in the GPCR-mediated signaling transduction, has been linked to cancer. GNB4 has been reported to be related to the prognosis in patients with urothelial bladder and colorectal carcinomas and shown to be involved in breast cancer cell growth (23, 33). In the present study, both ERK2 and GNB4 were upregulated in patients of anti-NMDAR encephalitis compared with healthy controls. In contrast, lncRNA NONHSAT084853.2 was downregulated. These data provide evidence that MAPK inhibition may represent a possible therapy for anti-NMDAR encephalitis. Negatively correlating lncRNAs, such as lncRNA NONHSAT084853.2, can be the subject of further in-depth investigation for their potential roles as diagnostic biomarkers and for their potential therapeutic effects. In addition, we validated the upregulation of mRNA GUCY1A3, IDS, PARVB, and TROVE2 in anti-NMDAR encephalitis via PCR. Among these, GUCY1A3 encodes the α1 subunit of soluble guanylate cyclase (sGC), which forms a heterodimeric enzyme with the β1 subunit encoded by GUCY1B3 (34). This complex serves as the primary receptor for nitric oxide (NO). NO binding to the heme iron of sGC induces cyclic guanosine monophosphate (cGMP) production, subsequently activating the cGMP-dependent protein kinase (PKG) pathway. Promoter activity analysis of GUCY1A3 identified consensus sequences for transcription factors including NFAT and NF-κB (p50) (35). Given that both NFAT and NF-κB are master regulators of inflammation, GUCY1A3 may be regulated by inflammatory signals, analogous to RNF213. Another key regulator is endothelial nitric oxide synthase (eNOS). Studies report GUCY1A3 upregulation by eNOS in pulmonary vasculature, and its mutations were initially detected in quasi-moyamoya disease (syndromic MMD) patients. TROVE2 is a highly conserved protein that binds and regulates the cellular distribution of small RNAs, particularly Y RNAs and rRNAs (36). Crucially, TROVE2 provides nucleic acid surveillance by binding retroelements such as Alu transcripts, which could otherwise drive inflammation, aberrant RNA editing, mutagenesis, and potential malignancies. Upon binding small RNAs, TROVE2-containing ribonucleoproteins (RNPs) may contribute to innate immune processes, including NLRP3 inflammasome activation—a mechanism closely linked to M2-phenotype polarization of macrophages (37). Furthermore, TROVE2 enhances anti-inflammatory effects through estrogen-induced macrophage polarization. As a well-established autoantigen, TROVE2 is implicated in multiple autoimmune diseases, including Sjögren’s disease (SjD), systemic lupus erythematosus (SLE), rheumatoid arthritis (RA), and systemic sclerosis (SSc), where anti-TROVE2 autoantibodies are frequently detected. However, the mechanisms underlying immune targeting of Ro60 (TROVE2) remain elusive. Our study provides foundational evidence for further investigation of these molecular pathways in anti-NMDAR encephalitis.

For the majority of patients with anti-NMDAR encephalitis, the triggers are often multifactorial and may not be readily identifiable. The diagnosis is mostly without preceding events of viral infections or tumorigenic events after regular check. Of note, even in those patients, it sometimes still presented an underlying predisposition toward metabolic and autoimmune aberrations (38–40), which is consistent with our GO and KEGG analysis. By comparing our results with those from others who performed tissue sequencing using the EAE mouse model, we found consistent findings in the GO enrichment related to immune system processes. Additionally, we identified further functional clusters involving cell communication, neurogenesis, and neuron projection. These results suggest that, beyond immune system functions, neuronal development and regeneration may also play important roles in anti-NMDAR encephalitis (41).

Although currently limited by the number of cases, many studies have conducted sequencing analyses with small sample sizes. In our study, sample size remains a limitation as well. In addition, one of limitation of our study is the lack of cerebrospinal fluid (CSF) data. Anti-NMDAR encephalitis is fundamentally a CSF-dominant disorder where intrathecal processes drive pathogenesis, and although the use of plasma for transcriptomic analysis is logistically more feasible—especially in pediatric or critically ill patients—it may not fully capture intrathecal immune responses or neuronal processes occurring within the central nervous system. Therefore, our findings should be interpreted with caution, as peripheral transcriptomic profiles may only partially reflect the disease-specific mechanisms in the brain. Future studies incorporating CSF samples will be critical for validating and extending these observations. What’s more, the majority of the annotation of lncRNAs in the co-expression network have not been examined with detailed experiments. Although the possible function of the lncRNAs can be hypothesized based on tentatively extrapolated from the mRNA expression data, in-depth and well controlled investigations, using both *in vitro* and *in vivo* techniques are needed to address their functions and assess their potential as diagnostic markers and as therapeutic targets. With such studies, advanced care of autoimmune encephalitis could be developed.

## 5 Conclusion

The current findings, for the first time, implicate the potential role of lncRNAs and mRNAs in pathogenesis of anti-NMDAR encephalitis, and hopefully lead to further understanding of genetic and epigenetic mechanism of anti-NMDAR encephalitis and the development of new diagnostics and therapeutics of this disorder.

## Data Availability

The datasets presented in this study can be found in an online repository. The name of the repository is GEO (https://www.ncbi.nlm.nih.gov/geo/), accession number is GSE305025.

## Footnotes

^1^ http://www.noncode.org

^2^ http://asia.ensembl.org/index.html

## Glossary

Anti-NMDAR: Anti-*N*-methyl-d-aspartate receptor
lncRNAs: long non-coding RNAs
mRNAs: messenger RNA
GO: Gene Ontology
KEGG: Kyoto Encyclopedia of Genes and Genomes
qRT-PCR: quantitative real-time polymerase chain reaction
ncRNAs: non-coding RNAs
miRNAs: micro RNAs
circRNAs: circular RNAs
SLE: Systemic lupus erythematosus
MAPK: Mitogen-activated protein kinase
MS: Multiple sclerosis
NMO: Neuromyelitis optica
RA: Rheumatoid arthritis
CNS: Central nervous system
EDTA: Ethylene diamine tetra acetic acid
RNA-Seq: RNA sequencing technology
FPKM: Kilobase of exon model per million mapped reads
FDR: False discovery rate
GAPDH: Glyceraldehyde phosphate dehydrogenase
